# Barriers to Seeking Mental Health Help in Saudi Arabia: A Systematic Review

**DOI:** 10.7759/cureus.60363

**Published:** 2024-05-15

**Authors:** Norah I Alhumaidan, Turki A Alotaibi, Khalid S Aloufi, Abdullah A Althobaiti, Nawaf Saleh A Althobaiti, Khaled Althobaiti, Wijdan A Almutiri, Khawlah Alhaqbani, Tafe Alboqami, Latifah Albeheiri, Njoud F Alfaisal

**Affiliations:** 1 College of Medicine, Princess Nourah bint Abdulrahman University, Riyadh, SAU; 2 College of Medicine, Taif University, Taif, SAU; 3 College of Medicine, Northern Border University, Arar, SAU; 4 College of Medicine and Surgery, Taif University, Taif, SAU; 5 Family Medicine, King Abdullah bin Abdulaziz University Hospital, Riyadh, SAU

**Keywords:** saudi arabia, help, seeking, mental health, barriers

## Abstract

Globally, mental disorders have become a significant burden, affecting a substantial number of individuals. Accessing mental health services is crucial for effective treatment and improving outcomes. However, significant barriers to seeking health services can impede access and contribute to the treatment gap. This systematic review aims to identify and analyze the perceived barriers to seeking mental health services in Saudi Arabia. A comprehensive search was conducted among four databases (PubMed, Web of Science, ProQuest, and Science Direct) to identify relevant studies published between 2018 and 2023. Studies that investigated barriers that could prevent psychiatric patients from seeking mental health services in Saudi Arabia were included. Data extraction and synthesis were performed to identify common themes and barriers. The review included a total of six studies that examined barriers to seeking mental health services in Saudi Arabia. The identified barriers encompassed a range of factors, including stigma, lack of awareness, concerns about confidentiality, limited availability of services, negative attitudes toward professional help, and cultural and religious beliefs. The lack of knowledge, as well as the negative attitude toward mental health care, was a perceived barrier to help-seeking in most studies. Furthermore, stigma was consistently reported as a predominant barrier, preventing individuals from seeking mental health care. This systematic review highlights the barriers to seeking mental health services in Saudi Arabia. Addressing these barriers is essential for improving access to mental healthcare and reducing the treatment gap. Strategies should focus on destigmatization efforts, increasing awareness, ensuring confidentiality and privacy, providing culturally appropriate care, and addressing structural limitations. By implementing these strategies, healthcare systems can improve access to mental health care and the overall well-being of individuals experiencing mental disorders in Saudi Arabia.

## Introduction and background

In recent years, the importance of seeking help for mental health concerns has gained widespread recognition as a crucial step toward accessing appropriate support and enhancing overall quality of life. However, despite deliberate efforts to reduce the stigma surrounding mental health, young individuals seem to display reluctance to seek assistance, particularly from general practitioners [1]. Emerging adults in Saudi Arabia struggle with a variety of mental health issues, such as anxiety, depression, and mood disorders related to identity exploration, unstable employment and relationships, and the period between adolescence and adulthood [2].

As highlighted by the World Health Organization, concerns about the mental health of young people are becoming increasingly prevalent. Notably, depression and suicide rank as the second and third most common causes of mortality among individuals aged 15 to 29, respectively [3]. Around two in five Saudi youth meet the criteria for a mental health condition during their lifetime, but only 5% of these people seek mental health services. [4]. Concerns pertaining to financial barriers, logistical challenges such as transportation or apprehensions regarding confidentiality, fears of others discovering their struggles, the perception that they possess the capability to manage the issue independently, and skepticism about the efficacy of the proposed treatment further contribute to the multifaceted array of factors impeding a person's willingness to seek help for mental health issues [5]. Moreover, a person waits longer than necessary to seek medical attention, which might worsen their illness and prevent it from being effectively treated. As a result, delaying seeking assistance increases the amount of time that a patient is unwell without receiving treatment, which has a detrimental effect on the effectiveness of treatment and is associated with a decline in overall health [6].

Conducting a comprehensive systematic review focused on examining the repercussions of mental health-related stigma on the inclination to seek help revealed discernible negative associations, indicating that individuals encountering stigma tend to exhibit reluctance or hesitation in seeking assistance for mental health issues [7]. The aims and scope of this systematic review are to identify and analyze existing literature on the barriers to seeking mental health help among individuals in Saudi Arabia, assess specific obstacles hindering the pursuit of professional mental health support in this population, and subsequently offer insights and recommendations to enhance mental health help-seeking behaviors in Saudi Arabia based on the systematic review's findings.

## Review

Methods

Literature Search Strategy

Preferred Reporting Items for Systematic reviews and Meta-Analyses (PRISMA) guidelines were followed in the conduct of this systematic review. PubMed, Web of Science, ProQuest, and Science Direct databases were searched extensively with no restrictions placed on the duration of the search. The search strategy used included these terms: ("Saudi Arabia" AND ("mental health" OR "seeking" OR "mental health support" OR "accessing psychological services") AND ("barriers" OR "obstacles" OR "challenges" OR "issues")) to identify all studies related to the barriers to mental health seeking in Saudi Arabia.

Inclusion and Exclusion Criteria

This systematic review included studies in English exploring barriers to seeking help for any mental health concern, which included individuals of all ages residing in Saudi Arabia, studies conducted in various settings, and all study designs except for review studies. Exclusion criteria included non-English papers that did not explore barriers to seeking care for any mental disorder, studies that included individuals residing in countries other than Saudi Arabia, and studies that were review studies.

Selection of Articles and Data Extraction

All the articles found from the primary search were imported to Rayyan for removal of duplication title screening. Four authors (T.A.A., K.S.A., A.A.A., and K.N.A.) independently screened based on title and then (N.I.A.) resolved outstanding disagreements and conflict by mutual consensus. Two independent authors (N.S.A. and W.A.A.) conducted the full-text screening. Extracted data from studies included study details, study design, participants’ characteristics, mental health disorders, if present, and barriers.

Risk-of-Bias Assessment

In this systematic review, the risk-of-bias assessment was conducted among non-randomized studies of interventions (NRSI). We used the ROBINs-1 tool to assess NRSIs [8]. Two independent reviewers (N.I.A. and T.A.A.) and one reviewer (K.A.A.) assessed the study's methodological quality and resolved conflicts by mutual consensus. The outcome assessed was the barriers to seeking mental health in 2018-2023. The judgment options were (low, moderate, serious, and critical) and the overall risk of bias was reached using signaling questions.

Results

**Figure 1:**
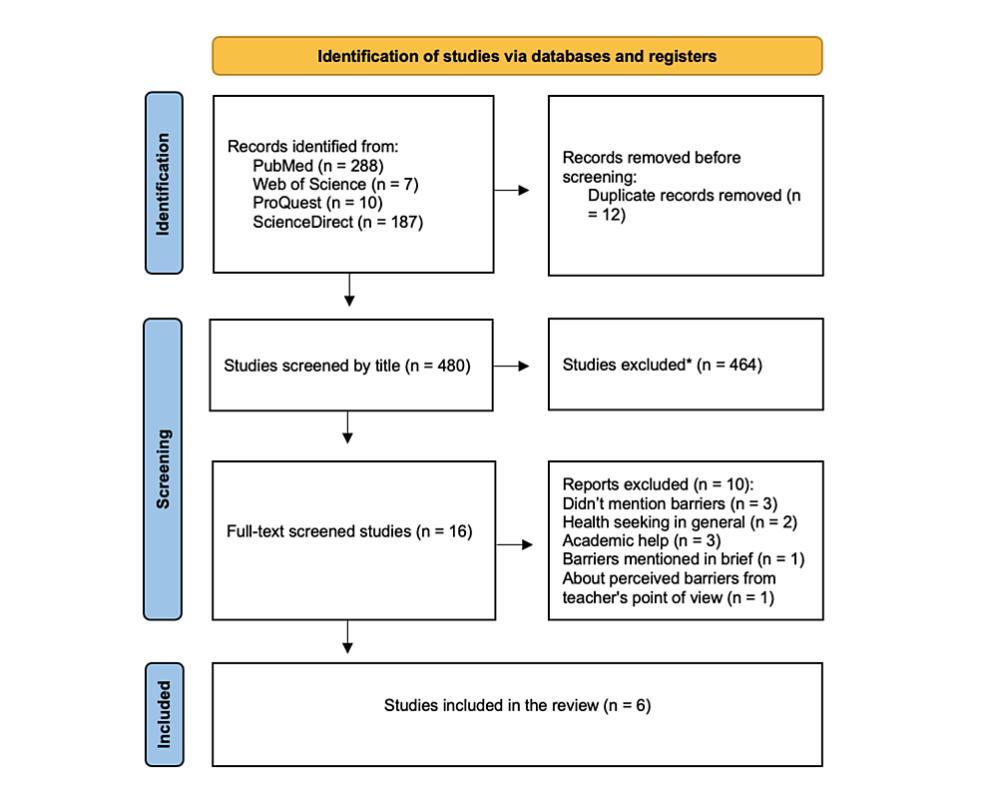
PRISMA flowchart depicting the selection of studies. PRISMA: Preferred Reporting Items for Systematic Review and Meta-Analysis. *Not eligible for our criteria.

Initially, 492 papers were extracted through four database searches (PubMed, Web of Science, ProQuest, and Science Direct), of which 12 were deleted as duplicates. Regarding the remaining 480 articles, 464 were excluded through title screening. Following full-text screening and assessment, 16 articles were excluded because they did not match the study's objective. Specifically, 10 papers were reports excluded, three did not mention barriers, two papers discussed health seeking in general, three papers were about academic help, one study concluded barriers briefly, and one was about perceived barriers from a teacher's point of view. Finally, six articles were included in the systematic review (Figure 1).

Study Characteristics

The characteristics of the studies of the perceived barriers of help-seeking regarding mental health are detailed in Table 1. The studies were published between 2018 and 2023 in Saudi Arabia. All studies were performed using qualitative methods (n = 6). Furthermore, the methodology employed involved face-to-face interviews in one study, and five studies depended on the survey method to collect data. The number of participants in the included studies varied from 12 to 5,644. All studies included both genders with a minimum age of 15 years. Most studies (n = 4) included samples not selected based on the subjects’ mental health status. However, two studies were conducted among participants with self-reported anxiety and depression with different grades in addition to mood disturbance, substance use, and impulse disorders. Several barriers to mental health seeking were discussed in the included studies, and stigma was the commonly reported barrier.

**Table 1:** Characteristics of the included studies and identified barriers to mental health seeking

No.	Study ID	Objective	Study design	Participants' characteristics	Mental disorders	Barriers to mental health seeking
1.	Alaqeel et al., 2023, Riyadh, Saudi Arabia [9]	To explore the factors that could prevent students from seeking mental health services	Cross-sectional questionnaire-based study	Number: 434, age: 15-29 years. Gender: male: 72.1%, female: 27.9%	Anxiety and depression with different grades	1. Feeling that my disorder is not an important issue (44%). 2. Worries about others not being able to comprehend my issues (37.2%). 3. Challenges in accessing appropriate care (32.4%). 4. Concerns about confidentiality (31.9%). 5. Belief that seeking help implies personal weakness (22.2%). 6 Fear of having mental health records documented on academic records (20.3%). 7. Fear of unwanted intervention (18.4%). 8. Lack of availability of services (15%). 10. Stigma of mental health care (14%).
2.	Noorwali et al., 2022,Saudi Arabia [4]	To examine the factors that hinder or facilitate the utilization of mental health care among young adults in Saudi Arabia	A qualitative research study was conducted using semi-structured interviews.	Number: 12, age: 21-24 years. Gender: male: 16.7%, female: 83.3%	Not available	1. Public stigma. 2. Lack of awareness. 3. Unprofessional mental health physicians. 4. Limited accessibility to services and information. 5. Lack of support from families. 6. Intrapersonal dilemmas. 7. Misconceptions influenced by religious beliefs.
3.	Alissa NA, 2021, Saudi Arabia [10]	To examine the knowledge regarding social barriers associated with mental health among adults	A cross-sectional study	Number: 1,632, age: >18 years. Gender: male: 17.5%, female: 82.5%	Not available	1. Social barriers may prevent the patients from seeking mental health help (78.9%). 2. Stigma (76.3%). 3. Culture (61.5%). 4. Negative perceptions (56.2%). Among males, culture was reported as the most prevalent social barrier affecting their willingness to seek help for mental health issues, with a percentage of 72.0%. Stigma was the second most commonly identified barrier, with 70.1%, followed by negative perceptions at 62.3%. For women, stigma was the most frequently mentioned barrier, with a percentage of 67.7%. Culture ranked second at 60.3%, and negative perceptions were identified as the third most prevalent barrier, with 59.7%.
4.	Alsahali S, 2021, Saudi Arabia [11]	To assess the attitudes of pharmacy students towards individuals with mental illness and their willingness to seek help for mental health issues, as well as their understanding of the causes of mental illnesses	A cross-sectional questionnaire-based study.	Number: 330, age: 19-25 years. Gender: male: 40.3%, female: 59.7%	Not available	There was a negative attitude regarding seeking help for mental disorders: 1. A sense of embarrassment at the prospect of their friends finding out that they were seeking professional help for an emotional disorder (48.8%). 2. They did not feel comfortable talking about personal problems with the psychiatrist (60%). 3. They did not perceive professional help as effective (15.5%).
5.	Alangari et al., 2020, Saudi Arabia [12]	To identify the obstacles faced by individuals with common mental illnesses in initiating and maintaining treatment	Study using data from Saudi National Mental Health Survey (SNMHS), a nationally representative study of the KSA population	Number: 711, age: 15–65 years. Gender: male: 38.9%, female: 61.1%	Mood disturbance, anxiety, substance use, and impulse disorders.	1. Low perceived need (50.7%). 2. Attitudinal barriers (48.8%): preferred to manage their condition independently (82.0%), perceived ineffectiveness (9.4%), stigma (4.7%), thought would get better (8.7%), and thought that their illness was not severe (13.5%). 3. Structural barriers (5.1%): financial (7.3%), availability (8.9%), transportation (5.8%), and inconvenience (6.4%).
6.	Mahmoud MA, 2018, Saudi Arabia [13]	To investigate the knowledge, attitudes, and perceptions of the Saudi population regarding mental illness and mental health services and to explore the barriers that hinder individuals' willingness to seek psychiatric help	Cross-sectional study	Number: 5,644, age: more than 20 years. Gender: male: 49.5%, female: 50.5%	Not available	1. Feel ashamed when visiting a psychiatrist (14.8 %). 2. They did not know about services provided by mental health facilities in Saudi Arabia (87.3%).

**Figure 2:**
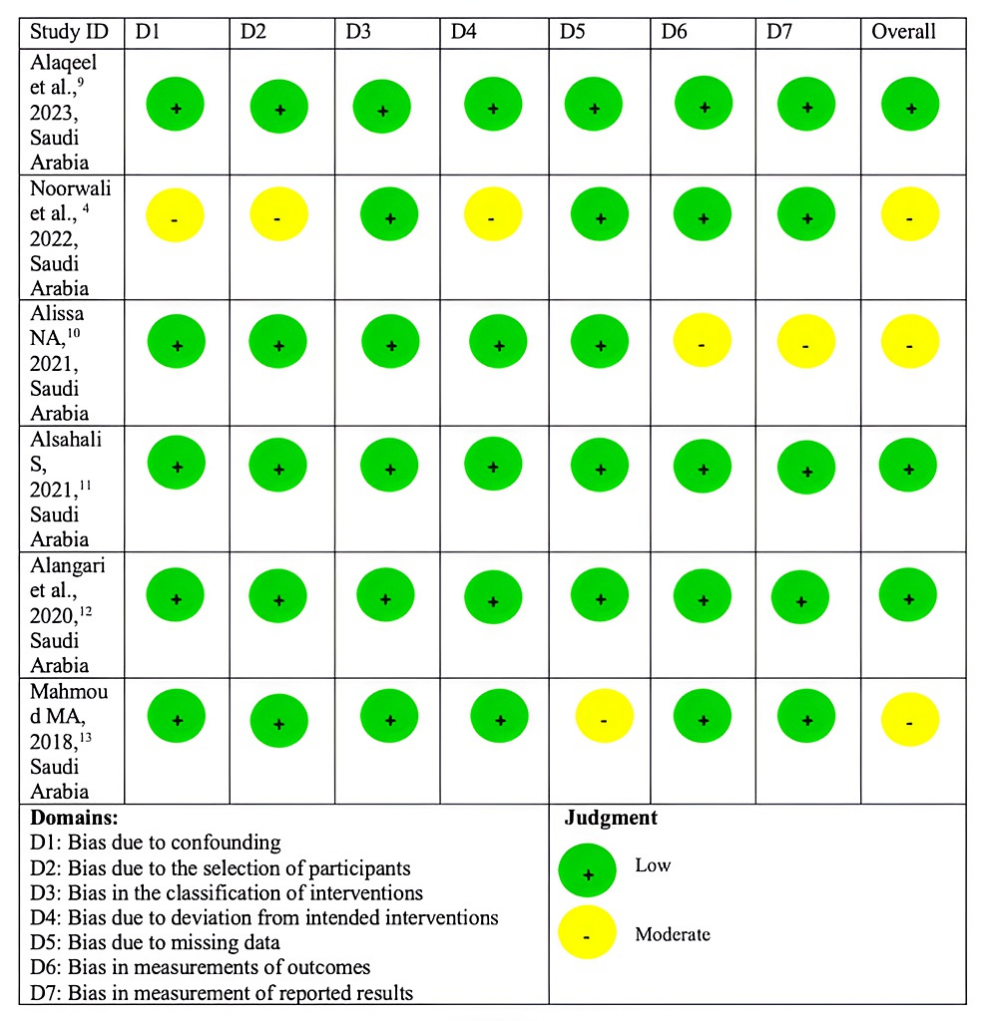
Robvis traffic light plot figure

As shown in Figure 2, the risk of bias revealed the overall quality of the included studies. Given the low and moderate risk of bias observed within our included studies, our findings suggest the need for more studies that focus on strategies that assess stigma, enhance awareness, maintain confidentiality, and identify structural limitations in healthcare systems.

Discussion

Globally, mental disorders are increasingly becoming a significant burden, affecting a high number of people. It was estimated that about one billion individuals are currently experiencing mental health or substance use disorders [14]. Failing to treat these mental disorders and terminating treatment prematurely not only increases the likelihood of chronic impairment, poor quality of life, and reduced educational achievements but also imposes substantial economic and societal challenges [15-17]. Moreover, it significantly contributes to escalated healthcare utilization and costs [18]. Recognizing and understanding these obstacles to treatment is essential in developing, designing, and enhancing access to mental health services [19].

The present systematic review indicated a range of perceived barriers to medical help-seeking in Saudi Arabia. The included studies highlighted a range of barriers to seeking mental health services in Saudi Arabia, including stigma, lack of awareness, concerns about confidentiality, limited availability of services, and negative attitudes toward professional help. 

It was estimated that beliefs about mental illness may alter patterns of help-seeking and response to treatment among patients [20]. In the present review, the lack of knowledge and negative attitude about mental health services was a perceived barrier to help-seeking [4,9-12]. These findings are consistent with previous reviews [21-23]. This lack of knowledge about available mental health resources and services indicates the need for widespread education campaigns.

Mental stigma is a major issue that affects several aspects of the patient’s life and the prognosis of the disease. It is estimated to prevent patients with mental disorders from seeking care and delay treatment and recovery. In addition, it may lead to isolation and unemployment among those patients [24-26]. According to the mentioned studies, stigma to mental health illness was the most predominant barrier regarding seeking mental health help. A study by Alissa [10] found that a high proportion of the participants (76.3%) reported that the stigma prevented them from mental health care. Moreover, the other three studies indicated that participants reported stigma as a barrier to medical care [4,8,11]. On the other hand, feeling ashamed of visiting a psychiatrist and embarrassed if friends knew of getting professional help for an emotional disorder were other two reasons that could prevent them from seeking medical care [10,12]. Our results are consistent with a systematic review that found that stigma and embarrassment were the most prominent barriers to help-seeking for mental health problems [22].

To a lesser extent, two articles highlighted the issue of confidentiality concerns as participants reported a lack of confidentiality [8] and discomfort in discussing personal problems with psychiatrists [10]. Their concerns may be attributed to the fear of defecting their image or reputation. Our results are similar to a prior systematic review that determined barriers to mental health services among Arab adults [27].

Furthermore, cultural and religious beliefs were barriers to help-seeking. A study by Alissa [10] found that 61.5% of the participants indicated cultural barriers hindered them from mental healthcare. Another study found that misconceptions based on religious beliefs were a significant barrier to seeking mental health care. The influence of cultural factors emerges as barriers, highlighting the importance of tailoring mental health services to cultural contexts to ensure they are acceptable and accessible.

Structural barriers were reported in two studies [8,12] including financial barriers, availability of services, difficulty with access to care, transportation issues, and inconvenience. Inadequate accessibility and availability of mental health services may exacerbate existing barriers, particularly in a context where individuals may already be reluctant to seek help.

It is important to acknowledge the limitations of the reviewed studies. The number of included studies is relatively small, and the findings are specific to the context of Saudi Arabia. Therefore, the results may not be generalizable to other regions or populations. In addition, the studies relied on self-reported data, which may be subject to recall or social desirability biases.

## Conclusions

This review found various barriers to seeking mental health care, such as stigma, lack of awareness, confidentiality concerns, limited service availability, negative attitudes toward professional help, and cultural/religious beliefs. Stigma, in particular, was a predominant barrier reported by participants, preventing them from seeking mental health care. Addressing the barriers to seeking mental health services is essential for improving access and reducing the treatment gap. Healthcare systems can improve access to mental health services and enhance well-being by targeting stigma, increasing awareness, ensuring confidentiality, and addressing structural limitations.
